# Rights of the Medical Staffs of Hospitals and the College of Physicians of Philadelphia

**Published:** 1870-01

**Authors:** 


### Rights of the Medical Staffs of Hospitals and the College of Physicians of Philadelphia.

Many of our readers are aware that the clinical lectures of the Pennsylvania Hospital have been interfered with by the Board of Managers having unexpectedly authorized female students to attend them This innovation occurred after the male students had purchased their tickets of admission to the practice of the house, and was regarded by them, as well as by the medical staff, as being in contravention of a contract implied in the sale and purchase of the tickets, as a measure which threatened greatly to diminish the value of the clinical lectures.

Upon the latter ground, also, a protest against it was presented at a general meeting of the physicians and surgeons attached to the several city hospitals, and was signed by them and a number of leading practitioners.

Moved by a similar sentiment the College of Physicians has not only pronounced upon the principles involved in the particular case which suggested the judgment, but also, as becomes a body so distinguished by its age, learning, and professional rank, has grounded its conclusions upon a just sense of the dignity of the profession, and its right to have a voice in the discussion of all questions which affect the relations of physicians and surgeons with the charitable institutions which they both serve and support.

It is one of the anomalies of the present age that medicine, which was never before so full of knowledge and power, nor so lavish in conferring benefits upon society, should, nevertheless, be so generally decried by teachers of philosophy, and subjected to such wanton insults by those it has most efficiently served.

In the army and navy, not only of the United States, but of Europe also, the claims of the medical staff to an appropriate rank are treated by the political authorities with derision and contempt. The wise and enlightened counsel of our brethren to Boards of Managers concerning the construction and economy of hospitals is unceremoniously thrust aside; their administration of medical and surgical offices is sometimes interfered with, and they are made to feel, as far as can be, that, not educated, scientific, and skillful physicians, but managers and trustees, albeit entirely destitute of professional knowledge, are the proper and rightful judges of medical questions, and are entitled to the power, which in fact they exercise, of over-riding remonstrance and despising advice. This conduct appears the more extraordinary, as it is certainly the most offensive, when it is remembered that in this country physicians serve the hospitals gratuitously, while everywhere else their services are remunerated.

The reasons for these anomalies, it appears to us, are two in number. The one is that physicians, by unselfishly giving their time and skill to public institutions, have cheapened their value. The other is that the superficial and illogical education, which even the lowest classes of society now obtain, has created a class of critics and judges whose dogmatism is on a par with their ignorance, and whom no fear of disastrous consequences deters from rash experiment and arbitrary innovation.

It seems, therefore, to be high time that whatever of manliness and independence there is in the medical profession should be aroused, and that the managers of hospitals should be told, respectfully but plainly, that they are not the owners in fee simple of these institutions, but only administer them in trust for the public who support them, for the sick to whom they are devoted, for the physicians without whom they could not exist, and for the students of medicine to whose education they are indispensable.

This statement will enable our readers to understand more clearly the subjoined resolutions, which were unanimously adopted at an unusually large meeting of the College of Physicians of Philadelphia, held Dec. 1, 1869, and ordered to be published:

"*Resolved*, That as hospitals depend in a great measure for their efficiency and good repute upon the character and skill of their medical officers, and as these officers habitually perform their laborious and often dangerous duties without compensation and with great devotion and zeal; it would seem that justice, as well as courtesy, required that in all things pertaining to the medical discipline of such institutions, the medical staff should not only be consulted, but that no measure materially affecting the patients should be adopted without their concurrence.

"*Resolved*, That in our judgment such consultation and concurrence are equally desirable whenever it is proposed by the governing board of a hospital to change the system, established by long usage and general consent, of giving clinical instruction; and that students of medicine authorized to attend the clinics, as well as the medical staff, have good reason to feel aggrieved by regulations which are innovations upon established custom, which affect their interests seriously, and which have been enacted without their knowledge, concurrence and consent"

JOHN H. PACKARD, *Secretary.*

